# Calcium supplementation for the prevention of hypertension: a synthesis of existing evidence and implications for practise

**DOI:** 10.12968/bjca.2023.0010

**Published:** 2024-02-24

**Authors:** O Hamer, A Mohamed, Z Ali-heybe, E Schnieder, JE Hill

**Affiliations:** University of Central Lancashire; Blackpool Teaching Hospitals NHS Foundation Trust; Blackpool Teaching Hospitals NHS Foundation Trust; Liverpool University Hospitals NHS Foundation Trust; University of Central Lancashire

**Keywords:** Systematic review, Hypertension, Calcium, Dietary supplementation, Blood pressure

## Abstract

Hypertension (also known as high blood pressure), is a medical condition characterized as a persistently raised blood pressure of the pulmonary artery. Effective interventions to treat hypertension typically involve two approaches: lifestyle modifications and pharmacotherapy. One specific lifestyle intervention which aims to increase calcium uptake through dietary supplementation, has recently gained popularity because of its potential to be low-cost and population based. Research suggests that this intervention may be effective given that calcium has been found to have an inverse relationship with blood pressure and hypertension. That said, studies have shown that there may be potential risks to patient health through adverse events such as kidney stone formation and increased cardiovascular events. Association between calcium supplementation and adverse events could have an impact on population health and prevent widespread adoption of the intervention. Because of the need to establish the effectiveness of this intervention assessed against any possible harms, it is now necessary to review the current evidence and evaluate its implications for clinical practise.

## Introduction

Hypertension (also known as high blood pressure) is a medical condition characterized as a persistently raised blood pressure of the pulmonary artery (Pokharel et al. 2022). The condition is typically defined as a systolic blood pressure greater than 139 mm Hg and/or a diastolic blood pressure greater than 89 mm Hg (Pokharel et al. 2022). Hypertension is estimated to affect around 1.13 billion people globally, with approximately one in three adults experiencing the condition (Zhou et al. 2021). There are two main categories of hypertension: (1) primary hypertension, also known as essential hypertension (which accounts for the majority of cases); and (2) secondary hypertension (Oparil et al. 2018). Primary hypertension is thought to be caused by a combination of genetic, environmental, and lifestyle factors (e.g., poor dietary intake and physical inactivity) (Tziomalos 2020). Secondary hypertension is typically caused by underlying medical conditions such as kidney disease, sleep apnoea, adrenal gland tumours, thyroid dysfunction and some medications (e.g., decongestants) (Tziomalos 2020).

According to the World Health Organization, hypertension is a primary risk factor for cardiovascular disease (Whitworth and Chalmers 2004). It is also a major contributor to the global burden of disease, as it increases the risk of heart attack, stroke, and kidney failure (Oparil et al. 2018; Zhou et al. 2021). There are several factors that can increase an individual’s risk of developing hypertension which include age, family history, obesity, smoking, excessive alcohol consumption, high salt intake, low potassium intake, lack of physical activity, and chronic stress (Filippini et al. 2017; Lesniak and Dubbert 2001; Liu et al. 2017; Midgley et al. 1996; Mills et al. 2020; Neter et al. 2003; Nordmann et al. 2011). Many of these risk factors are modifiable through lifestyle changes, such as maintaining a healthy diet, regular physical activity, smoking cessation, and low alcohol consumption (Mills et al. 2020).

Effective interventions to treat hypertension typically involve two approaches: lifestyle modifications and pharmacotherapy (Carey et al. 2018; Valenzuela et al. 2021). Pharmacotherapy has previously been established as an effective intervention for hypertension, reducing blood pressure with medications such as thiazide diuretics, calcium channel blockers, angiotensin-converting enzyme inhibitors, angiotensin receptor blockers and beta-blockers (Carey et al. 2018). Lifestyle modifications such as maintaining a healthy diet, regular physical activity, avoiding smoking and excessive alcohol consumption have also been shown to be effective in reducing blood pressure (Mills et al. 2020; Valenzuela et al. 2021). One specific lifestyle intervention which aims to increase calcium uptake through dietary supplementation, has recently gained popularity because of its potential to be low-cost and population-based (Cormick and Belizán 2019; Cormick et al. 2022). Research suggests that this intervention may be effective given that calcium has been found to have an inverse relationship with blood pressure and hypertension (Dickinson et al. 2006). That said, studies have shown that there may be potential risks to patient health through adverse events such as kidney stone formation and increased cardiovascular events (Bolland et al. 2008; Cormick and Belizán 2019). There is now a need to establish the effectiveness of this intervention assessed against any possible harms, and evaluate its implications for clinical practise.

## Aim of commentary

This commentary aims to critically appraise the methods used within the review Cormack et al, 2022 and expand upon the findings in the context of clinical practice.

## Methods

The Cochrane systematic review employed a comprehensive search strategy including three databases and three trial registries from inception to September 2020. Searches were conducted with no restrictions of language, publication year or publication status. Further to the database searches, bibliographies of included studies were screened for any unidentified trials. The systematic review included published, unpublished, and ongoing trials that evaluated the effects of dietary calcium intervention, such as supplementation or food fortification, on normotensive individuals of different ages (excluding pregnant women). The review included randomized designs, excluding quasi-randomized designs and the second phase of crossover trials. Calcium interventions included pills, tablets, or powder, as well as food or beverage fortification, compared to placebo or control. Studies with no placebo or control and those where calcium was combined with other macro or micronutrients were excluded. Screening and data extraction were conducted independently by pairs of review authors (cross checking them). Three reviewers independently assessed the methodological quality of the identified trials, using the criteria outlined in the Cochrane Handbook. Any disagreement was resolved through discussion with the whole author team.

The primary outcomes were hypertension, systolic blood pressure, diastolic blood pressure, and secondary outcomes were adverse events, withdrawals due to adverse events, kidney stone formation, iron deficiency anaemia, total mortality, cardiovascular events, myocardial infarction, stroke and sudden death. Meta-analysis was initially conducted using a fixed-effect model when it was reasonable to assume that studies were estimating the same treatment effect. In addition, meta-analysis using a random-effects model was also conducted when substantial statistical heterogeneity was detected.

## Results

High quality evidence suggests that calcium supplements can help to very slightly reduce both systolic (-1.37 mmHg 95% CI: -2.08 to -0.66) and diastolic blood pressure (-1.45 mmHg 95% CI: -2.23 to -0.67). There was also some evidence of a dose-response in that there was no evidence of effect for doses less than 1000 mg a day (moderate quality evidence). However, there was a statistically significant small reduction in systolic blood pressure for both doses ranging from 1000 mg to 1500 mg a day (-1.05 mmHg 95% CI: -1.95 to -0.19, high quality evidence) and 1500 mg a day or more (-2.79 mmHg 95% CI: -4.71 to -0.86, moderate quality evidence).

There was no evidence that calcium was statistically significantly more or less effective in people greater (moderate quality evidence) or less than 35 years of age (high quality evidence) for reducing systolic blood pressure. There was no evidence of a difference for the potential moderating factors of gender, duration of supplementation and supplementation type (fortification and supplementation). A range of sensitivity analyses found no evidence that removal of high risk of bias studies, industry-funded studies, position of measurement, less than 3.5 months of intervention, measurement method and clinic blood pressure made a statistically significant difference to the effects of calcium on blood pressure. Only one study measured side effects with no reported side effects and two further studies indicated that calcium supplementation was well-tolerated.

## Commentary

Using the AMSTAR-2 critical appraisal tool for systematic reviews, a total of 16 criteria out of 16 were judged to be satisfactory (Table 1) (Shea et al. 2017). Thus, it was judged that the systematic review provided an accurate and comprehensive summary of the evidence from existing studies available in the literature.

The National Health Service recommends that adults aged 19 to 64 should consume 700 mg of calcium daily (National Health Service, 2020). In the United Kingdom the mean calcium intake has been predicted to be above this threshold in people (Public health England, 2020). Based upon the findings of the review this dose level would not produce the effect levels identified in this review. Minimum doses of 1000 mg to 1500 mg a day are required to begin to see very slight reductions (-1.05 mmHg) in systolic blood pressure for people with normotensive blood pressure. This effect may be slightly increased with a larger dose of greater than 1500 mg per day, but the precision of this estimate is wide and there is no significant difference between these two doses. Furthermore, the NHS recommend that doses over 1500 mg per day may cause gastric irritation (National Health Service, 2020). Despite this review finding no adverse events, a previous reviews in this area highlights that calcium supplementation can increase the risk of hypercalcemia, constipation, excessive abdominal cramping, bloating, upper gastrointestinal events, gastrointestinal disease and gastrointestinal symptoms (Lewis et al, 2012; Walker 2022). Therefore, it is important to note that when prescribing large doses, additional gastric issues may occur.

This review highlights when using calcium supplementation there is currently little evidence that the moderating factors of gender, duration of supplementation and supplementation type make a difference on its effectiveness on blood pressure. Despite this review finding no evidence of difference in the method of supplementation, there is still much debate on the possible impact that supplement versus dietary calcium intake may have on other cardiac risk factors (Yang et al 2020). Furthermore, calcium intake through dietary methods may provide a more consistent and frequent delivery of calcium which may result in more efficient bone building (Booth et al 2012). However, if dietary intake of calcium cannot be achieved other supplement sources may be required. Calcium supplementation varies widely with supplements ranging from 110 to 600 mg in stand-alone supplementation and can be 500 or 600 mg in calcium plus vitamin D (Supplementation Office of Dietary Supplements 2023). It is important to note when using combination supplements such as calcium and vitamin D, that this may have additional effects in regard to increasing the risk of hypercalcemia (Walker 2022). Specific calcium contents for current commercialised calcium supplementation can be found on the Dietary Supplement Label Database (https://dsld.od.nih.gov/).

**Figure:**
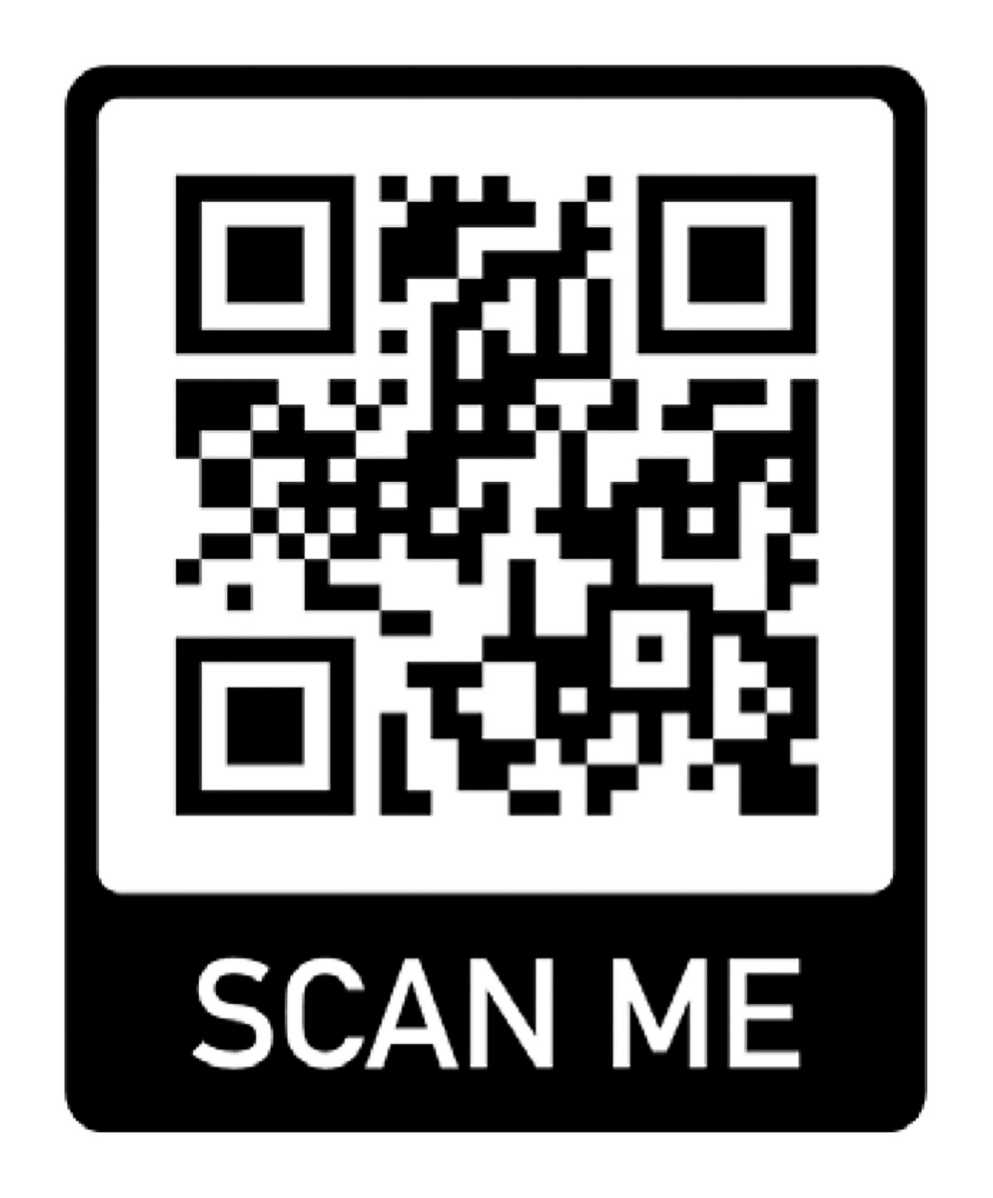
Dietary Supplement Label Database

It is essential to inform the patient that non-prescribed calcium supplementation will come with a financial cost, and that this should be discussed before making the recommendation. Calcium intake can be increased through dietary adaptations by increasing the intake of foods such as Milk, Yogurt, Tofu, Turnip greens, and Sardines canned in oil with bones (Office of the Surgeon General. 2004).

Due to the wide variation in effects identified within the potential moderating factors seen in this review, further high-quality random controlled trials are required exploring the effects of gender, duration of supplementation and supplementation type. This review revealed that there is a scarcity of studies that investigate the effects of blood pressure in young adults and children. Consequently, further exploration of this subpopulation is warranted. Furthermore, further research is required on the exact mechanisms of the effect of calcium on lowering blood pressure. A particular focus should be placed on reporting any adverse events in future studies, as there was inconsistent reporting of adverse events in the included studies of this review.

## CPD reflective questions

- What factors should be considered when making the recommendation to use calcium supplementation?
- Why else should you take calcium supplementation?
- Which foods are high in calcium?

## Figures and Tables

**Table 1:** Critical appraisal using the AMSTAR-2 tool for assessing systematic reviews

AMSTAR 2 items	Responses
1. Did the research questions and inclusion criteria for the review include the components of PICO?	Yes – The study outlined the participants, intervention, comparator and outcomes in the methods section.
2. Did the report of the review contain an explicit statement that the review methods were established prior to the conduct of the review and did the report justify any significant deviations from the protocol?	Yes – The protocol was registered on the Cochrane Database of Systematic Reviews.
3. Did the review authors explain their selection of the study designs for inclusion in the review?	Yes - Studies included all published, unpublished and ongoing trials with random allocation to dietary calcium intervention versus placebo or control.
4. Did the review authors use a comprehensive literature search strategy?	Yes - Electronic searches of three databases and three trial registries were included.
5. Did the review authors perform the study selection in duplicate?	Yes – Study selection was independently conducted by pairs of reviewers.
6. Did the review authors perform data extraction in duplicate?	Yes - Data extraction was conducted by pairs of reviewers who checked the other’s extracted data.
7. Did the review authors provide a list of excluded studies and justify the exclusions?	Yes - The authors provided reasons for exclusion and listed the studies in an appendix.
8. Did the review authors describe the included studies in adequate details?	Yes – A characteristics of included studies table was available in an appendix.
9. Did the review authors use a satisfactory technique for assessing the risk of bias in the individual studies that were included in the review?	Yes - Three reviewers independently assessed the methodological quality of the identified trials.
10. Did the review authors report on the sources of funding for the studies included in the review?	Yes – The authors did report funding sources for each study where the data was available and acknowledge the importance in the sensitivity analysis.
11. If meta-analysis was performed did the review authors use appropriate methods for statistical combination of results?	Yes - Meta-analysis was conducted with appropriate methods using fixed and random effects models for statistical combination of the results.
12. If meta-analysis was performed did the review authors assess the potential impact of RoB in individual studies on the results of the meta-analysis or other evidence synthesis?	Yes - The study conducted a sensitivity analysis to assess the potential impact of bias in individual studies on the results of the meta- analysis.
13. Did the review authors account for RoB in individual studies when interpreting/discussing the results of the review?	Yes – The authors discussed the results in relation to the quality of evidence (i.e., moderate to high quality trials).
14. Did the review authors provide a satisfactory explanation for and discussion of, any heterogeneity observed in the results of the review?	Yes – The authors explored heterogeneity within each meta-analysis.
15. If they performed quantitative synthesis did the review authors carry out an adequate investigation of publication bias (small study bias) and discuss its likely impact on the results of the review?	Yes – A funnel plot in figure 4 suggested that the findings of this systematic review showed no evidence of publication bias.
16. Did the review authors report any potential sources of conflict of interest, including any funding they received for conducting the review?	Yes - The authors reported no competing or conflicting interests.

## Key Points

1. High quality evidence suggests that calcium supplements can help to slightly reduce systolic blood pressure.
2. High quality evidence also suggests that calcium supplements can help to modestly reduce diastolic blood pressure.
3. There is some evidence of a dose-response.
4. There was no evidence that calcium supplementation was linked to any side effects, albeit few studies reported any adverse events.

## References

[R1] Cormick G, Belizán JM (2019). Calcium Intake and Health. Nutrients.

[R2] Booth A, Camacho P (2013). A Closer look at calcium absorption and the benefits and risks of dietary versus supplemental calcium. Postgrad Med.

[R3] Falaschetti E, Mindell J, Knott C, Poulter N (2014). Hypertension management in England: a serial cross-sectional study from 1994 to 2011. Lancet.

[R4] Houston MC, Harper KJ (2008). Potassium, magnesium, and calcium: their role in both the cause and treatment of hypertension. J Clin Hypertens (Greenwich).

[R5] Huang L, Chu Y, Huang X (2020). Association between gene polymorphisms of voltage-dependent Ca2+ channels and hypertension in the Dai people of China: a case-control study. BMC Med Genet.

[R6] Lewis JR, Zhu K, Prince RL (2012). Adverse events from calcium supplementation: relationship to errors in myocardial infarction self-reporting in randomized controlled trials of calcium supplementation. J Bone Miner Res.

[R7] National Health Service (2020). Calcium, minerals and vitamins.

[R8] Office of the Surgeon General (US) (2004). Bone Health and Osteoporosis: A Report of the Surgeon General.

[R9] Public health England (2020). Official Statistics NDNS: results from years 9 to 11 (combined) – statistical summary.

[R10] Shea BJ, Reeves BC, Wells G, Thuku M, Hamel C, Moran J, Moher D, Tugwell P, Welch V, Kristjansson E (2017). Amstar 2: A critical appraisal tool for systematic reviews that include randomised or non-randomised studies of healthcare interventions, or both. BMJ.

[R11] Supplementation Office of Dietary Supplements (2023). Dietary Supplement Label Database.

[R12] Walker MD, Shane E (2022). Hypercalcemia: A Review. JAMA.

[R13] Yang C, Shi X, Xia H, Yang X, Liu H, Pan D, Sun G (2020). The Evidence and Controversy Between Dietary Calcium Intake and Calcium Supplementation and the Risk of Cardiovascular Disease: A Systematic Review and Meta-Analysis of Cohort Studies and Randomized Controlled Trials. J Am Coll Nutr.

